# Terabyte capacity-enabled (10 x 400 Gbps) Is-OWC system for long-haul communication by incorporating dual polarization quadrature phase shift key and mode division multiplexing scheme

**DOI:** 10.1371/journal.pone.0265044

**Published:** 2022-03-10

**Authors:** Sushank Chaudhary, Lunchakorn Wuttisittikulkij, Jamel Nebhen, Abhishek Sharma, Demostenes Zegarra Rodriguez, Santosh Kumar

**Affiliations:** 1 Wireless Communication Ecosystem Research Unit, Department of Electrical Engineering, Faculty of Engineering, Chulalongkorn University, Bangkok, Thailand; 2 College of Computer Science and Engineering, Prince Sattam bin Abdulaziz University, Alkharj, Saudi Arabia; 3 Guru Nanak Dev University, Amritsar, India; 4 Department of Computer Sciences, Federal University of Lavras, Lavras, Minas Gerais, Brazil; 5 School of Physics Science and Information Technology, Liaocheng University, Liaocheng, China; Nanchang University, CHINA

## Abstract

Inter-satellite optical wireless communication (Is-OWC) links can become promising solutions to realize the next-generation high-speed communication services. The operation of Global Navigation Satellite Systems can be improved with the use of Is-OWC links through ranging and communication services. However, the key challenge in Inter-satellite link (ISL) is its effective range which is limited due to pointing errors. In this work, we propose to develop a high-capacity and long-reach Is-OWC link by incorporating hybrid mode division multiplexing (MDM) and wavelength division multiplexing (WDM) schemes to transmit ten independent channels over 40000kms Is-OWC link. Each channel is capable of carrying 400Gbps data which is encoded by the dual polarization quadrature phase shift key technique with required signal to noise ratio (SNR) and received power. The proposed Is-OWC link satisfies the enhanced communication within Geostationary Earth Orbit (GEO) and Low Earth Orbit (LEO) satellites. The proposed Is-OWC is further evaluated under the impact of space turbulences, particularly transmitter and receiver pointing errors. The result reported that the proposed Is-OWC link can transmit 4Tbps data over 16000kms with the transmitter pointing error of 2μrad and receiver pointing error of 1μrad.

## I Introduction

**Fig 1:**
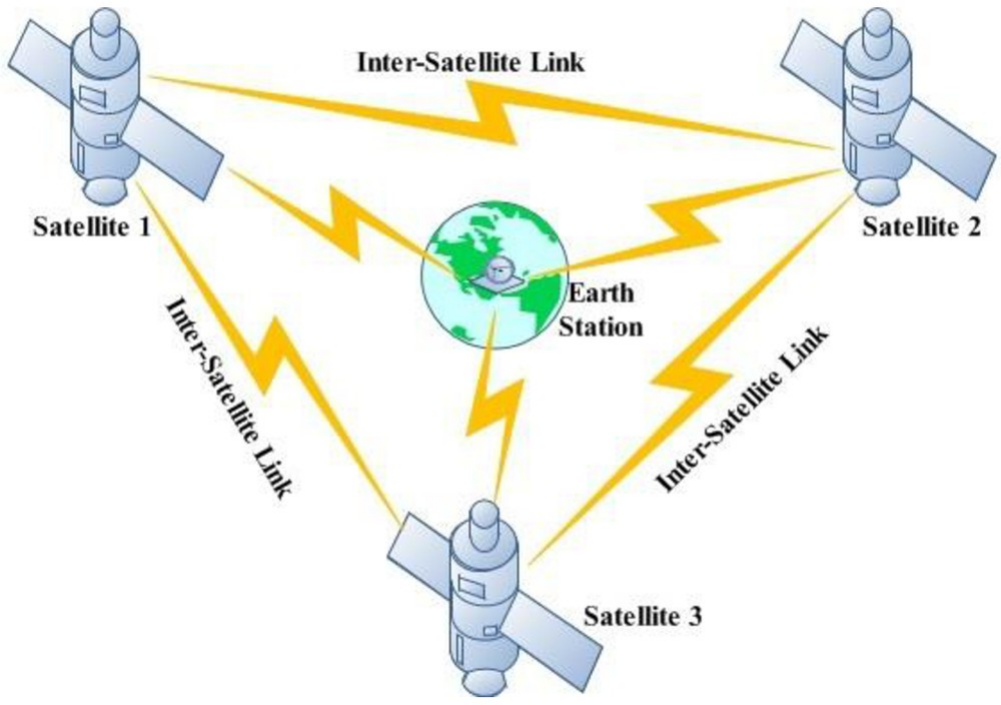
Is-OWC link.

In 1962, laser technology was first employed in communication systems largely in space communication to deliver global coverage for information broadcast [1]. Subsequently, quite a lot of government agencies, individual researchers and research institutes have achieved remarkable advancement in deep space and inter-satellite communication. For communication purpose, three geostationary satellites can cover the entire earth separated at 120° apart in free space [2]. A typical satellite link is shown in Fig 1. In 2003 [3], an inter-satellite optical wireless communication (Is-OWC) link was successfully demonstrated for the first time in which 50*Mbps* of data were transmitted between two satellites named as Advanced Relay and Technology Mission Satellite (ARTEMIS) and Satellite Pour I’observation de la Terre 4 (SPOT-4).

Is-OWC link deployed in low earth orbits (LEO) can also fulfil the demand of future wireless communication systems. Moreover, the 3^rd^ Generation Partnership Project (3GPP) is developing the techniques to integrate satellites into 5G and 6G networks [4–6]. The Is-OWC link offers many advantages including profuse unlicensed spectrum, resistance to electromagnetic and radio frequency interferences, high-speed and high-efficiency links, small infrastructure, low input power requirements and large bandwidth [7, 8]. However, the Is-OWC link uses high-frequency carrier signals that lead to narrow beam divergence angle which makes acquisition, tracking and pointing very difficult and hence leads to loss of information. Thus, pointing errors are considered more severe in Is-OWC as long-haul transmission makes it difficult to maintain line-of-sight communication. Further, any mechanical vibration, electronic disturbance or performance jitter adds to the pointing errors and ultimately degrades the system performance [9]. Many researchers have incorporated wavelength division multiplexing and on-off key modulation schemes to increase the capacity of Is-OWC links. In 2015 [10], authors investigated the performance of the Is-OWC system by employing a DWDM scheme. They compared performance of the return to zero (RZ) and non-return to zero (NRZ) encoding schemes by transmitting 10*Gbps* data over 5000*kms* Is-OWC link. In 2016 [2], authors incorporated the polarization interleaving scheme with WDM for enhancing the performance of the Is-OWC system. In that work, six independent NRZ-encoded channels, assigned as even and odd channels with X polarization to even channels and Y polarization to odd channels, with each one carrying 20*Gbps*, are transported over ISL of 1000*kms*. The reported results with clear eye patterns and acceptable SNR show successful transmission of 120*Gbps* data under the influence of space turbulences. In another work [11], authors compared modified duo binary return to zero (MDRZ), duo binary return to zero (DRZ) and carrier suppressed rerun to zero (CSRZ) modulation formats by using a WDM scheme to transmit 64 channels carrying 10*Gbps*, 20*Gbps* and 40*Gbps* data over an ISL range of 250*kms*. In 2018 [12], authors compared advance modulation schemes, particularly alternate mark inversion (AMI), chirped return to zero and differential phase shift keying (DPSK) modulation, by transmitting 64 channels over 3000*km* Is-OWC link. In [13–15], authors reported the use of advance modulation schemes for designing the Is-OWC link. In our recent work [16], we have demonstrated the use of hybrid spectral amplitude coding optical code division multiple access (SAC-OCDMA) and polarization division multiplexing (PDM) schemes for transmitting 100*Gbps* data over 25000*kms* under the impact of space turbulences. In another work [17], authors investigated dual polarization quadrature phase shift keying (DP-QPSK) modulation scheme to transmit 1.6*Tbps* data over the Is-OWC link of 20000*kms*. They also investigated the performance of their proposed IS-OWC link under the impact of space turbulences. In our other work in 2019 [18], we have demonstrated the Is-OWC link by employing DPSK with orthogonal frequency division multiple access (OFDM) scheme for the transmission of 10*Gbps* data over 20000*kms*. Whereas mode division multiplexing (MDM)–which is a prominent multiplexing scheme–has gained significant esteem in both wired and wireless optical systems from the last decade [19]. MDM can utilize Eigen modes for simultaneous data transmission over single wavelength optical carrier in order to improve system capacity in optical communications [20, 21]. In 2019 [22], authors reported comparative analysis of CSRZ, DRZ and MDRZ-QPSK modulation schemes for Is-OWC system by employing MDM scheme. They demonstrated the transmission of 40*Gbps* data over 4500*kms* Is-OWC link. In 2020 [23], authors reported the transmission of 80Gbps data over 6000*kms* Is-OWC link under the impact of pointing errors up to 2*μrad*. In this work, to further increase the transmission capacity as well as transmission range, we propose a high-capacity and long-reach Is-OWC system by employing hybrid MDM-WDM and spectral-efficient DP-QPSK schemes. DP-QPSK is mainly based upon digital signal processing (DSP) technology and offers high spectral efficiency and dispersion tolerance [24]. Our results show the successful demonstration of transmission of ten channels, with each carrying 400*Gbps* DP-QPSK encoded data, over ISL link of 40,000*kms* which no other previous work to the authors’ best knowledge reported. Furthermore, we have investigated the performance of the proposed Is-OWC link under space turbulences, particularly pointing errors (transmitting and receiving). The structure of the paper includes system modelling of the proposed Is-OWC link in Section II, its observations and discussions in section III, and overall findings in Section IV.

## II Hybrid DQPSK-MDM-WDM-Is-OWC modelling

**Fig 2:**
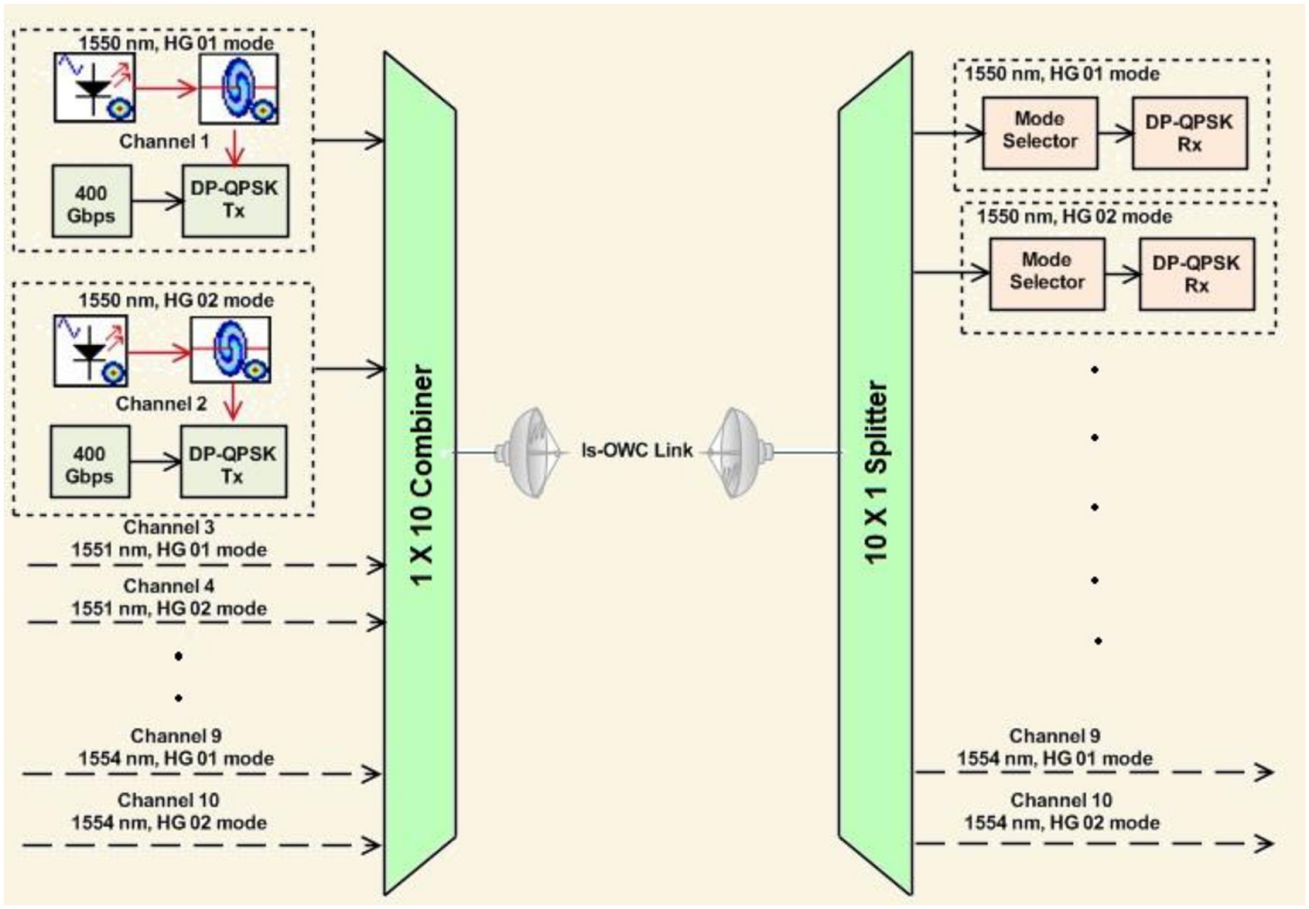
Proposed 10 × 400 Gbps WDM-MDM-DP-QPSKIs-OWC system.

The proposed DPQPSK-MDM-WDM system (400*Gbps* x10), modelled in OptiSystem^™^ software, is illustrated in Fig 2. The transmitter section consists of ten independent channels with each carrying 400*Gbps* DPQPSK-encoded data. The WDM scheme is implemented by using five different optical carriers with wavelengths starting from 1550*nm* to 1554*nm* whereas the MDM scheme is implemented by using two distinct Hermite Gaussian modes–HG*01* and HG*02*. Spatial (multimode) laser is used to generate two distinct HG modes. As shown in the Fig 2, the MDM scheme is integrated with the WDM scheme in such a manner that each wavelength can be used to transmit two channels by utilizing two modes (HG*01* and HG*02* modes).

**Fig 3:**
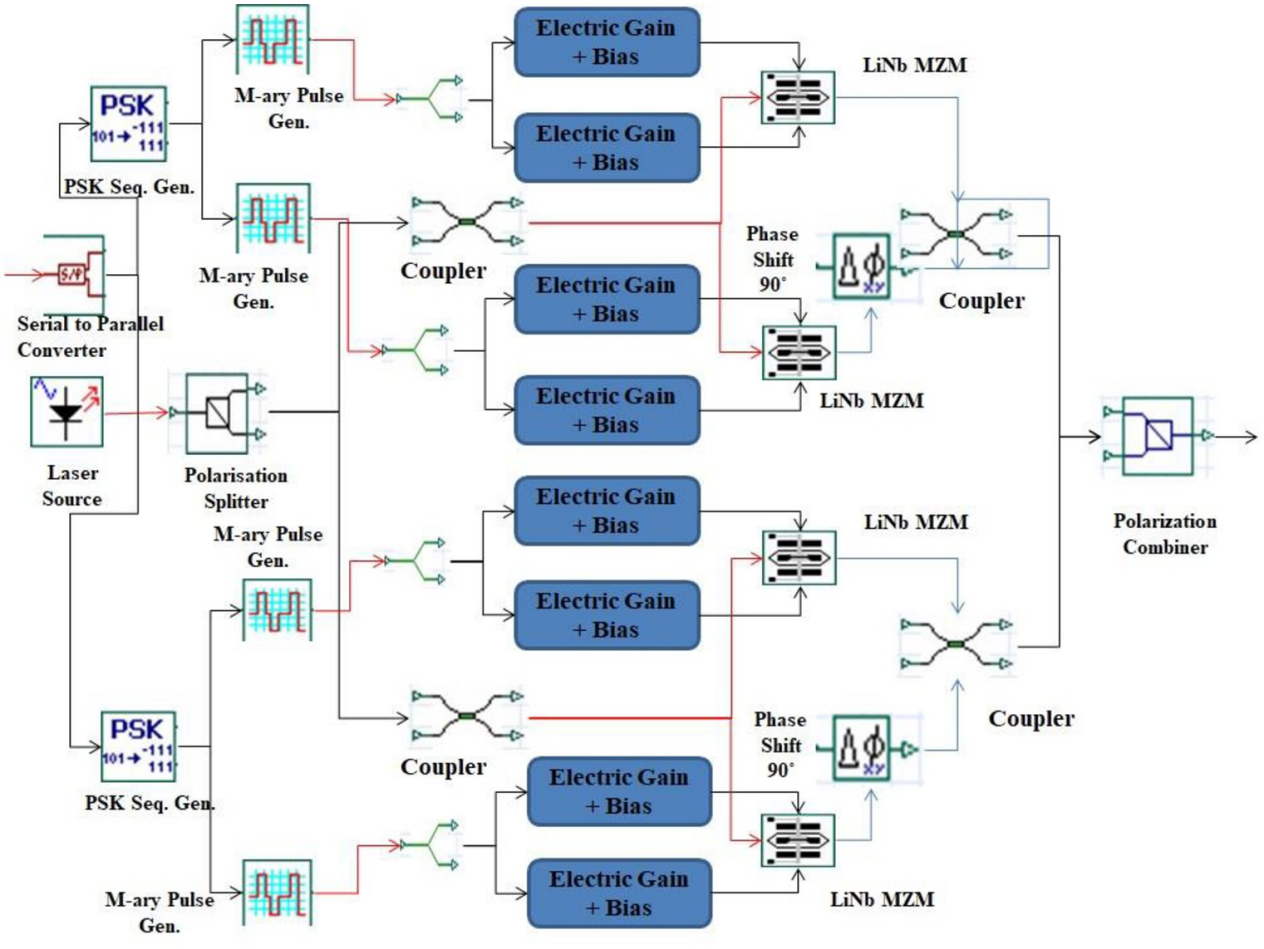
DP-QPSK transmitter unit.

**Fig 4:**
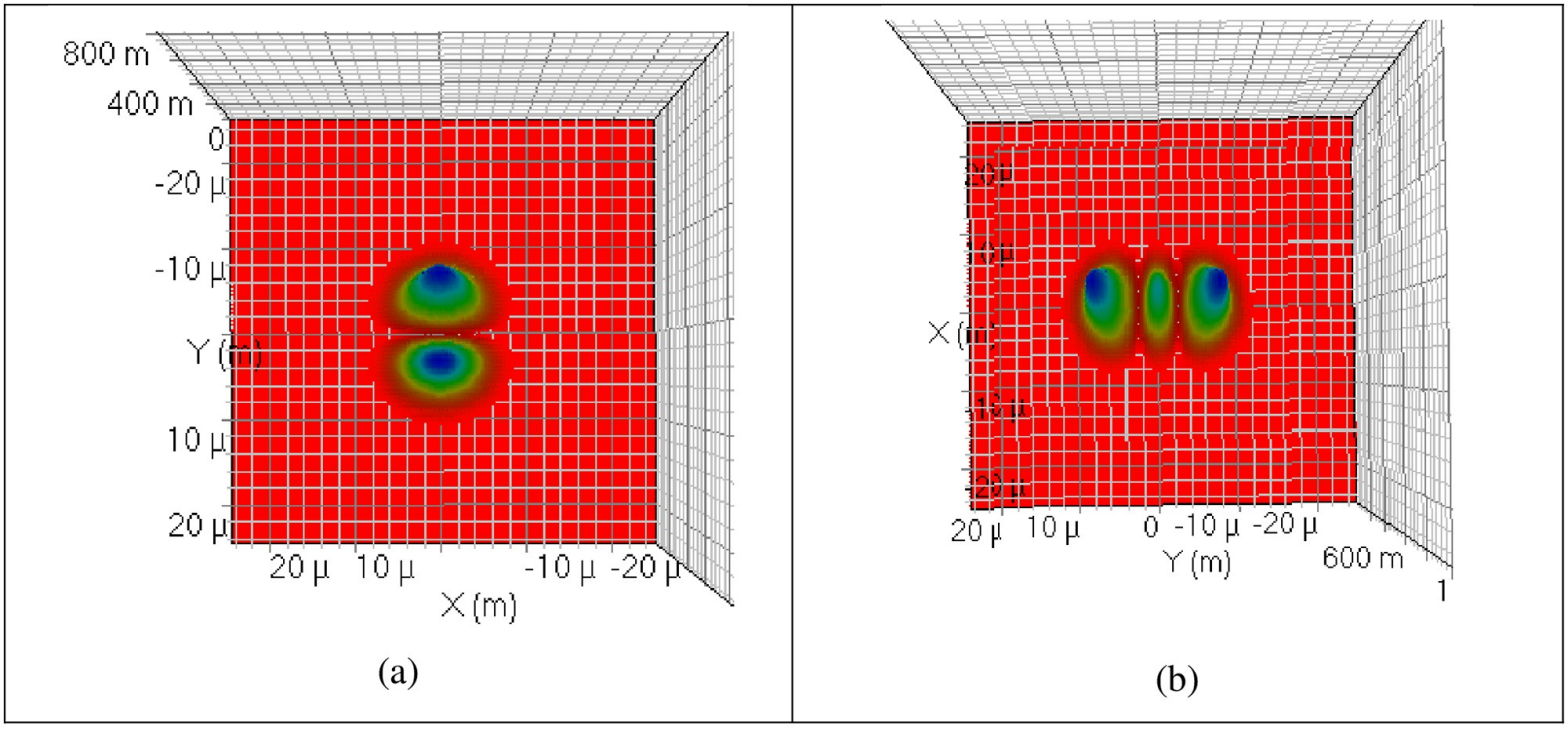
Excited modes at the transmitter (a) HG 00 mode (b) HG 01 mode.

In each channel, 400*Gbps* of data is encoded by using DP-QPSK encoder section and modulated over optical carrier generated by spatial laser. The DP-QPSK encoder consists of four dual-port Mechzender modulators (MZM) in order to attain the dual polarization quadrature carriers as shown in Fig 3. These modulators are derived from spatial laser with 10*dBm* of power and 10*MHz* of linewidth. This spatial laser generates relevant HG modes followed by vortex lens with a focal length of 10*mm* in order to attain the phase in modes. The HG modes can me described mathematically as:

ψm,nr,ϕ=Hm2xwo,xexp-x2wox2expjπx2λRox×Hn2ywo,yexp-y2woy2jπy2λRoy (1)

where, the X and Y indexes represent mode dependencies on their axes denoted by *m* and *n*, respectivel, *R*_*o*_ is the radius of curvature, *wo* is the spot size and *Hm* and *Hn* represents the Hermite polynomials. Fig 4 shows the HG 01 and HG 02 modes excited with the help of spatial laser.

Phase transformation is applied to transverse mode profiles by the vortex lens which can change the focus of the output beam. The applied phase transformation with this lens is given in Eq 1 [25]:

$$T\;\left(x,y\right)=-\text{exp}\;\;[-j\;\frac{\pi n\;\left(x^{2}+y^{2}\right)}{2\lambda f}+m\;\text{atan}\;\left(\frac{x}{y}\right)]$$ (2)

where *f* refers to the focal length, *m* is the vortex parameter and *n* refers to the refractive index.

These phase-transformed modes can be used to modulate 400*Gbps* DP-QPSK encoded data over optical light with the help of MZMs.

**Fig 5:**
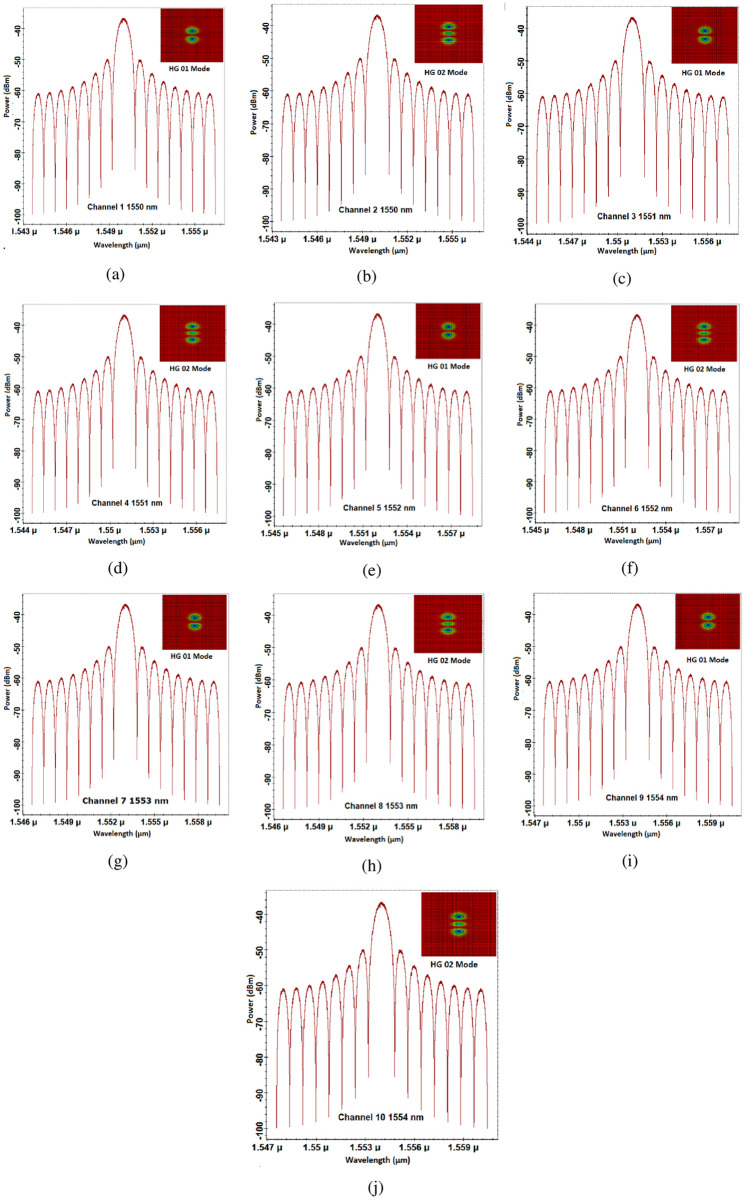
Optical spectrum at transmitter side (a) channel 1 (b) channel 2 (c) channel 3 (d) channel 4 (e) channel 5 (f) channel 6 (g) channel 7 (h) channel 8 (i) channel 9 (j) channel 10.

Thus, channels 1 and 2 are transmitted over 1550*nm* by using HG*01* and HG*02*, respectively. Similarly, channels 3 and 4 are transmitted over 1551*nm* by using HG*01* and HG*02* modes while channels 5 and 6 are transmitted over 1552*nm* by using HG*01* and HG*02* modes, channels 7 and 8 over 1553*nm* by using HG*01* and HG*02* modes, and channels 9 and 10 are transmitted over 1554*nm* by using HG*01* and HG*02* modes, respectively. Fig 5 shows the optical spectrum of each channel.

These MDM-WDM multiplexed DP-QPSK signals from different channels are integrated and transmitted over the Is-OWC link with a span of 40000*kms*. The channel modelling of Is-OWC can be defined as [16]:

$$P_{R}=P_{T}\eta_{T}\eta_{R}\;({\frac{\lambda }{4\pi Z})}^{2}\;G_{T}G_{R}L_{T}L_{R}$$ (3)

where *P*_*R*_ and *P*_*T*_ are the optical powers of received and transmitted signals respectively, *η*_*T*_ is the optical efficiency of the transmitter while *η*_*R*_ is the optical efficiency of the receiver. The wavelength of the optical carrier is represented by *λ*, and Is-OWC range can be defined by Z whereas *G*_*T*_ and *G*_*R*_ are the gains of transmitting and receiving antennas, respectively.

*L*_*T*_ and *L*_*R*_ denote the transmitter and receiver loss factors in Eq (2). The gains of transmitting antennas are given by [16]:

$$G_{T}=\pi D_{T}/\lambda^{2}$$ (4)

where *D*_*T*_ is the telescopic diameter of transmitting antenna. Similarly, the gain of the receiving antenna is given by the following equation:

$$G_{R}=\pi D_{R}/\lambda^{2}$$ (5)

where *D*_*R*_ is the telescopic diameter of the receiving antenna.

**Fig 6:**
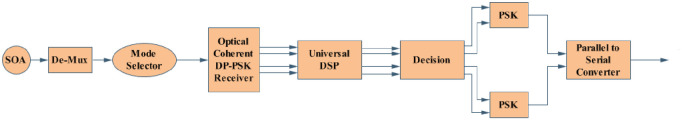
Receiver section of the proposed 10 × 400 Gbps WDM-MDM-DP-QPSKIs-OWC system.

**Fig 7:**
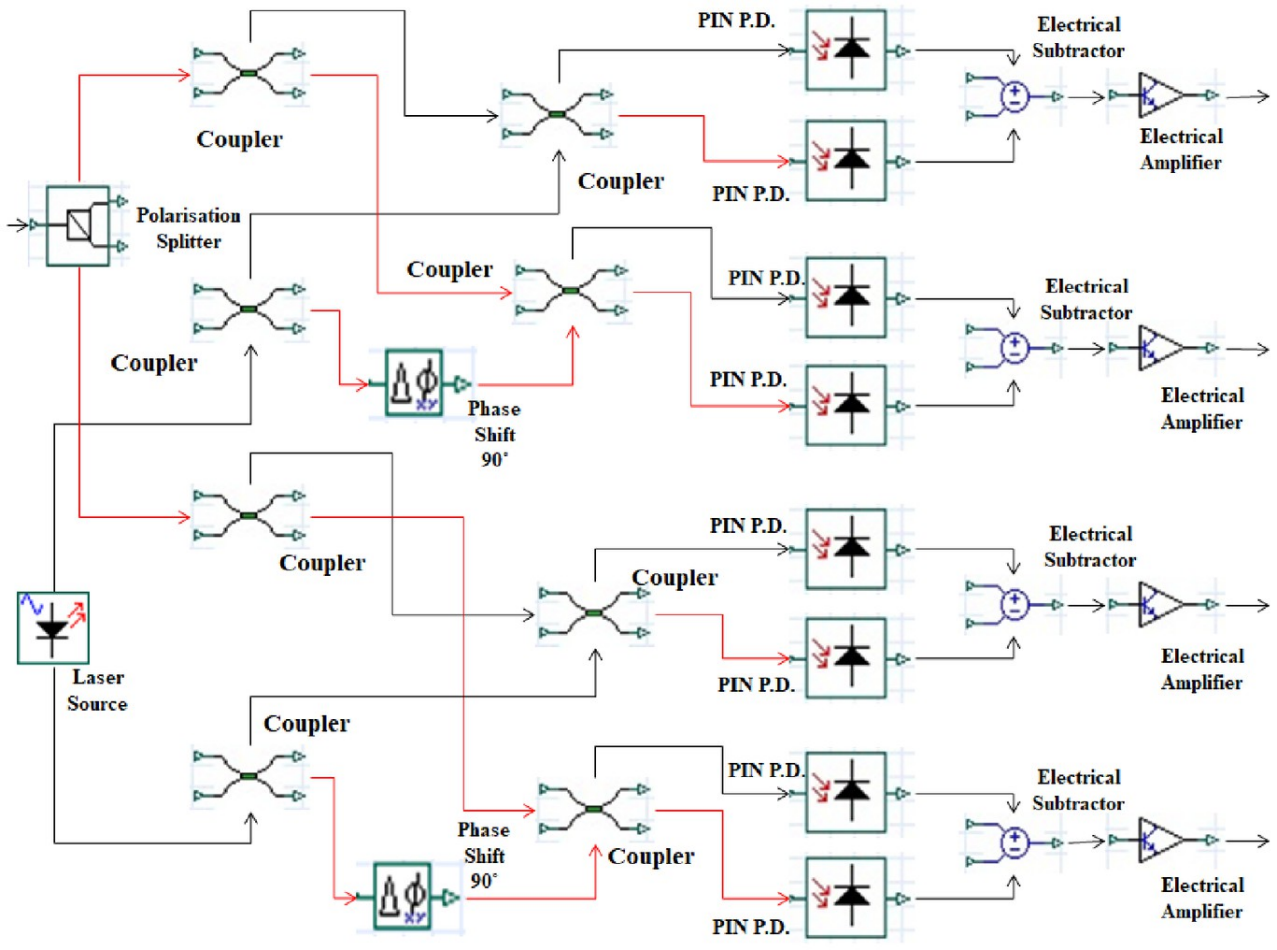
Optical coherent DP-QPSK receiver unit.

The loss factors for transmitter and receiver are further defined as [16]:

$$L_{T}=\text{exp}\;\left(-G_{T}\theta_{T^{2}}\right)$$ (6)

$$L_{T}=\text{exp}\;\left(-G_{R}{\;\theta }_{R^{2}}\right)$$ (7)

where *θ*_*T*_ and *θ*_*R*_ represent the angles of the transmitter and receiver pointing errors, respectively. Table 1 shows the simulation parameters of various components used in the proposed system. After transmitting through the Is-OWC link, the MDM-WDM-DQPSK encoded signals are fed to the semiconductor amplifier (SOA) with 0.12*A* of injection current for the post-amplification process. At the receiver side, the optical signal is de-multiplexed into the corresponding wavelengths with the help of the de-multiplexer. The output of the de-multiplexer is further fed to the mode selector filter which passes the relevant mode into the DQPSK receiver as shown in Fig 6. The DQPSK receiver consists of eight PIN photo diodes in order to attain the coherent QPSK detection. Fig 7 shows the internal architecture of optical coherent DP-PSK receiver.

**Table 1:** Modelling parameters of the proposed MDM-WDM-DPQPSK-Is-OWC system.

Component	Parameters	Value
Continuous Wave Laser	Spatial CW Laser power	-10*dBm*
	Laser Line width	10*MHz*
	Wavelength	1550*nm* to 1554*nm*
Data Rate	Number of Channels	10
	Data rate/channel	400 *Gbps*
Transmitter	Transmitter Pointing error angle	up to 5*μrad*
	Transmitter aperture diameter	150*mm*
Receiver	Receiver Pointing error angle	Up to 3*μrad*
	Receiver aperture diameter	300*mm*
Simulation window	Sequence Length	65536
	Sample per bit	4
Photo diode (PIN)	Responsivity	1*A/W*
	Dark Current	10*nA*
	Thermal noise power density	100e-024*W/Hz*
	Ionization ratio	0.9
	Vortex lens parameter	2
	Vortex lens focal length	10*mm*
	Amplifier injection current	0.15*A*
	Optical Confinement Factor	0.3
	Additional losses (pointing, synchronization etc.)	5*dB*

The output of the optical coherent DP-QPSK receiver goes into the universal DSP unit. The universal DSP components execute required functions and algorithms to assist in the recovery of original baseband information after the coherent detection. The DSP unit operates into two stages: pre-processing and post-processing. The pre-processing stage performs three functions which include noise addition to signal, DC blockage and normalization whereas the post-processing stage performs eight functions which include resampling, Down-sampling, Bessel filter, Timing Recovery, Nonlinear (NL) compensation, Quadrature Imbalance (QI) compensation, Chromatic Dispersion (CD) compensation, Adaptive Equalizer (AE), Carrier Phase Estimation (CPE) and Frequency Offset Estimation (FOE). The recovered signal is then sent to the decision unit that processes the electrical signals of *I* and *Q* received from the DSP Unit and normalizes the electrical amplitude based upon respective grid. Based on the normalized threshold settings, the DSP unit then conducts a decision on each received symbol. The output of this unit is then fed to a PSK sequence decoder in order to attain the original baseband information. Table 1 shows other modelling parameters of the proposed MDM-WDM-DPQPSK- Is-OWC system.

## III modelling results and discussion

**Fig 8:**
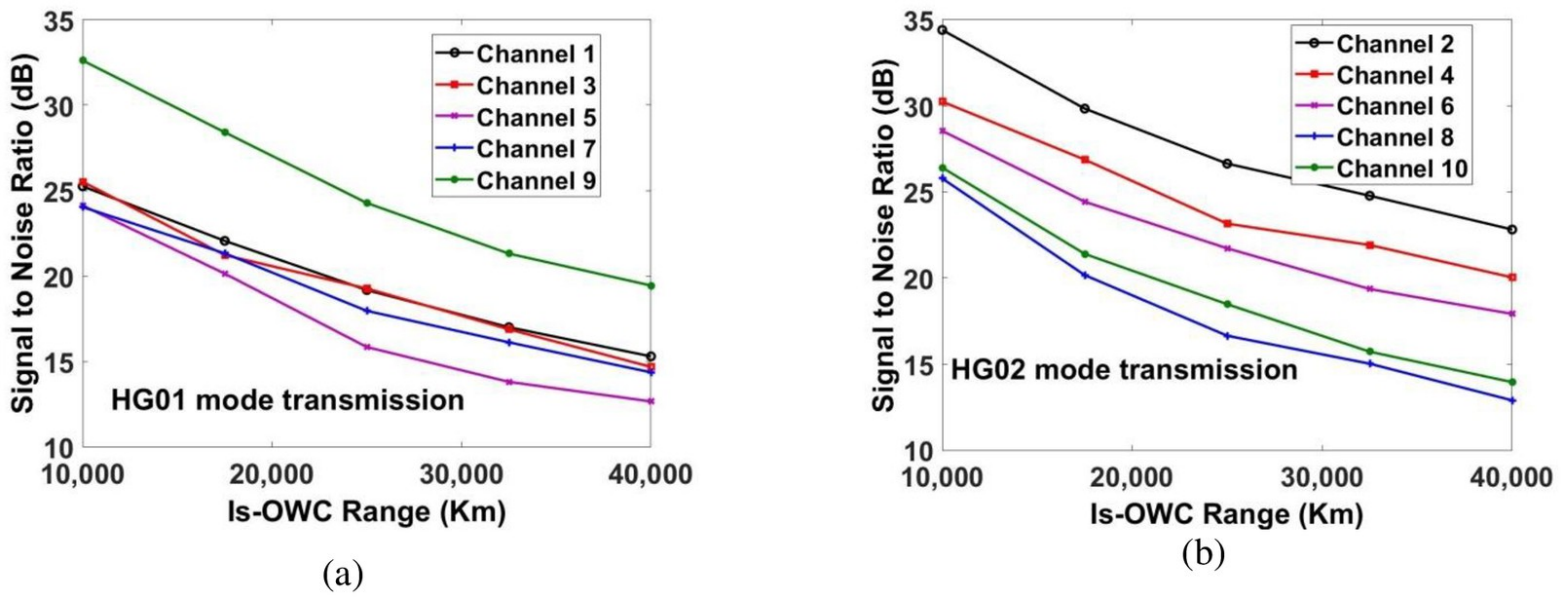
Evaluation of measured SNR for (a) channels 1, 3, 5, 7 and 9 for HG01 mode transmission and (b) channels 2, 4, 6, 8 and 10 for HG02 mode transmission.

**Fig 9:**
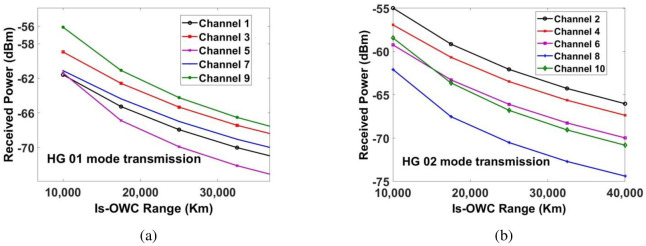
Measured power at output of receiver for (a) channels 1, 3, 5, 7 & 9 for HG01 mode transmission and (b) channels 2, 4, 6, 8 & 10 for HG02 mode transmission.

Signal to noise ratio (SNR) and received power are used for evaluating the performance of the proposed MDM-WDM-DPQPSK-Is-OWC system. In this section, we have discussed the observations from the modelling of the proposed Is-OWC system. Firstly, the Is-OWC link is assumed to operate in ideal conditions without considering any space turbulences. However, we have considered 5*dB* of additional geometrical losses. The measured SNR for the ten channels is shown in Fig 8. The channel which is transmitted by using HG*02* mode has higher SNR as compared to the channel transmitted by using HG*01* mode. In case of Channel 1 and Channel 2 (transmitted over 1550*nm*), an improvement in SNR ≈7*dB* is measured at Is-OWC link of 17500*kms* for Channel 2 as compared to Channel 1. In case of Channel 3 and Channel 4, an improvement of ≈5*dB* in SNR is measured for Channel 4 as compared to Channel 3 at the Is-OWC link of 17500*kms*. Similarly, at the same range, an improvement of ≈4*dB*, ≈1*dB* and ≈7*dB* is measured for channels 6, 8 and 10 as compared to channels 5, 7 and 9, respectively. The required acceptable SNR ≈20*dB* is achieved for all the channels up to 17500*kms*. Fig 9 shows the measurement of power of the recovered signal at the output of receiver. It shows that at the distance of 17500*km*, significant amount of power is received for all the channels in order to attain the required SNR≈20*dB*. However, while propagating through the Is-OWC link, HG*01* mode suffers more as compared to HG*02* mode. At the distance of 17500*kms*, the measurement of power of the recovered signal at the output of receiver is -65.26*dBm* for Channel 1, -59.13*dBm* for Channel 2, -62.58*dBm* for Channel 3, -60.66*dBm* for Channel 4, -66.88*dBm* for Channel 5, -63.27*dBm* for Channel 6, -64.36*dBm* for Channel 7, -67.51*dBm* for Channel 8, -61.09*dBm* for Channel 9 and -63.62*dBm* for Channel 10.

Table 2 shows the successful transmission of all channels carrying 4*Tbps* data up to 17500*kms* with the required SNR and power which shows significant improvement of the previous works. Now, in order to know the performance of the proposed Is-OWC system under the influence of space turbulences, we have applied pointing errors to the Is-OWC model. The range of the Is-OWC channel is fixed at 16000*kms*.

**Table 2:** Measured SNR and total received power without space turbulences.

Channel/Mode	SNR (dB) at distance (kms)					Total Received Power (dBm) at distance (kms)				
	10000	17500	25000	32500	40000	10000	17500	25000	32500	40000
Channel 1/ HG 01	25.24	22.05	19.17	17	15.29	-61.59	-65.26	-67.93	-70	-71.68
Channel 2/ HG 02	34.41	29.81	26.64	24.78	22.82	-54.97	-59.13	-62.05	-64.26	-66.02
Channel 3/ HG 01	25.51	21.22	19.26	16.27	14.67	-58.95	-62.58	-65.32	-67.43	-69.11
Channel 4/ HG 02	30.24	26.87	23.14	21.92	20.03	-56.92	-60.66	-63.46	-65.62	-67.35
Channel 5/ HG 01	24.12	20.13	15.83	13.80	12.65	-61.37	-66.88	-69.90	-72.09	-73.82
Channel 6/ HG 02	28.55	24.43	21.72	19.36	17.91	-59.22	-63.27	-66.09	-68.24	-69.97
Channel 7/ HG 01	24.03	21.32	17.95	16.11	14.36	-61.11	-64.36	-66.97	-69.04	-70.70
Channel 8/ HG 02	25.78	20.17	16.65	15.02	12.89	-62.07	-67.51	-70.50	-72.69	-74.38
Channel 9/ HG 01	32.61	28.41	24.27	21.30	19.44	-56.09	-61.09	-64.23	-66.52	-68.31
Channel 10/ HG 02	26.41	21.40	18.48	15.72	13.95	-58.42	-63.62	-66.78	-69.05	-70.81

**Fig 10:**
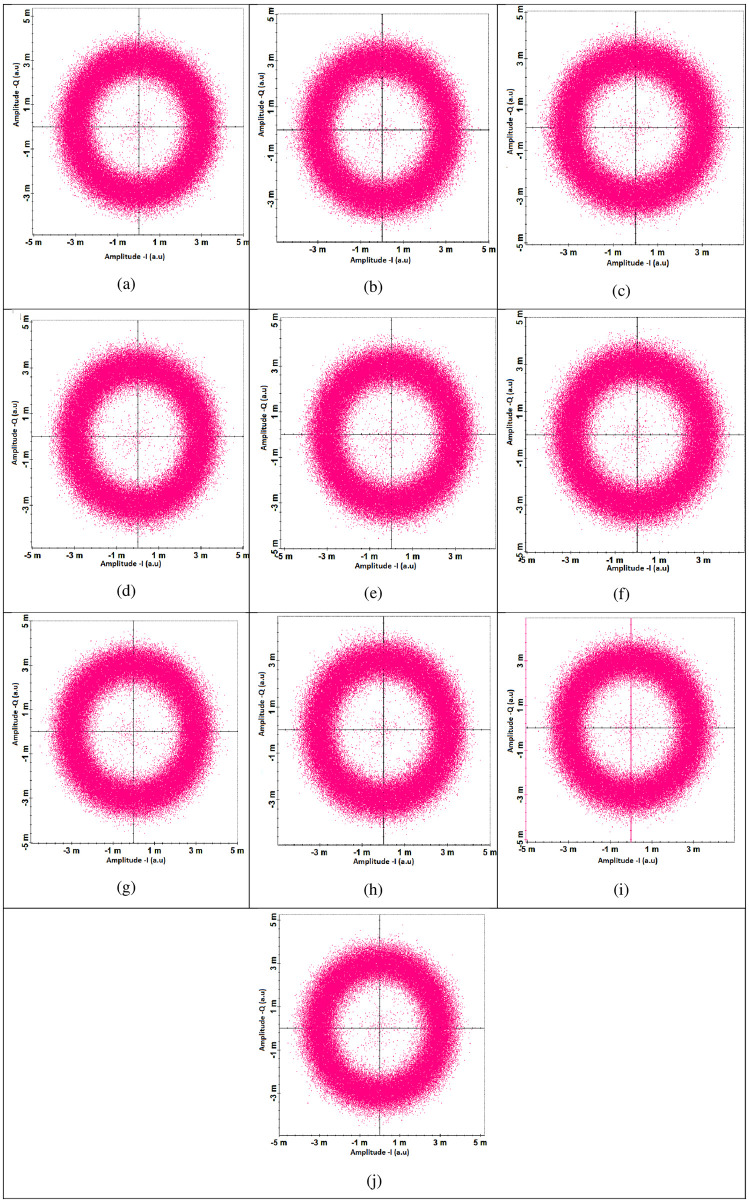
Measured constellations (17500 kms) at the receiver side before digital signal processing unit (a) channel 1 (b) channel 2 (c) channel 3 (d) channel 4 (e) channel 5 (f) channel 6 (g) channel 7 (h) channel 8 (i) channel 9 (j) channel 10.

**Fig 11:**
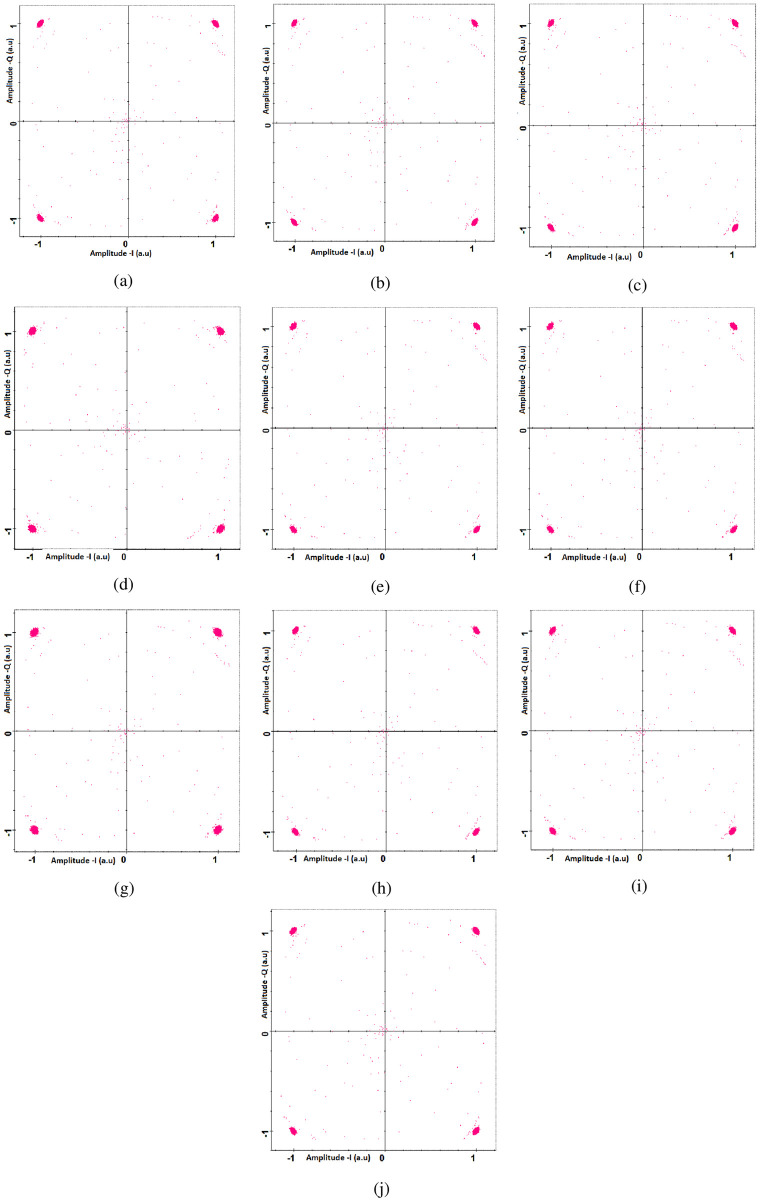
Measured constellations (17500 kms) at the receiver side after digital signal processing unit (a) channel 1 (b) channel 2 (c) channel 3 (d) channel 4 (e) channel 5 (f) channel 6 (g) channel 7 (h) channel 8 (i) channel 9 (j) channel 10.

**Fig 12:**
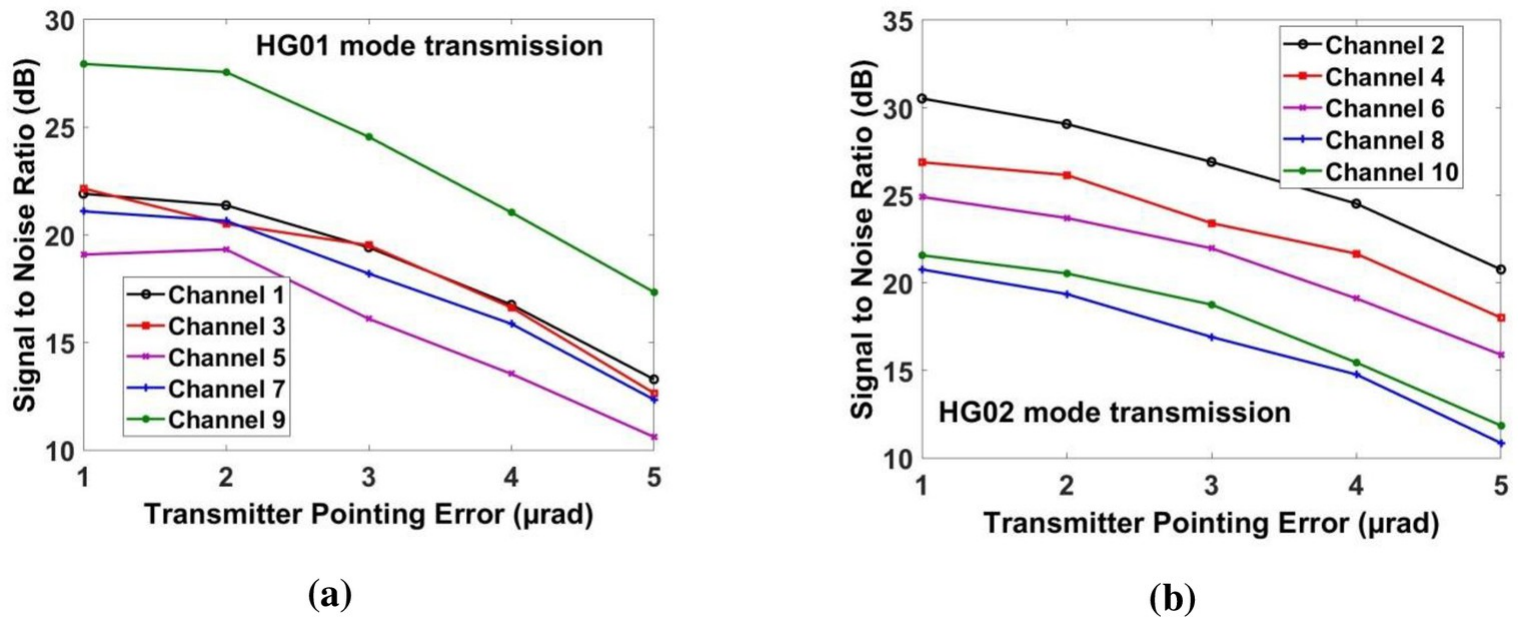
Evaluation of measured SNR under transmitting pointing error for (a) channels 1, 3, 5, 7 & 9 for HG01 mode transmission and (b) channels 2, 4, 6, 8 & 10 for HG02 mode transmission.

Figs 10 and 11 shows the measured constellations before and after DSP unit. It can be seen that DSP unit improves the constellations at the receiver side. Fig 12 shows the measured SNR for all the channels under the impact of transmitting pointing errors. It further shows that all ten channels can achieve the acceptable SNR≈20*dB* up to the transmitting error of ≈2.5*μrad* (beyond that SNR degrades the performance).

**Fig 13:**
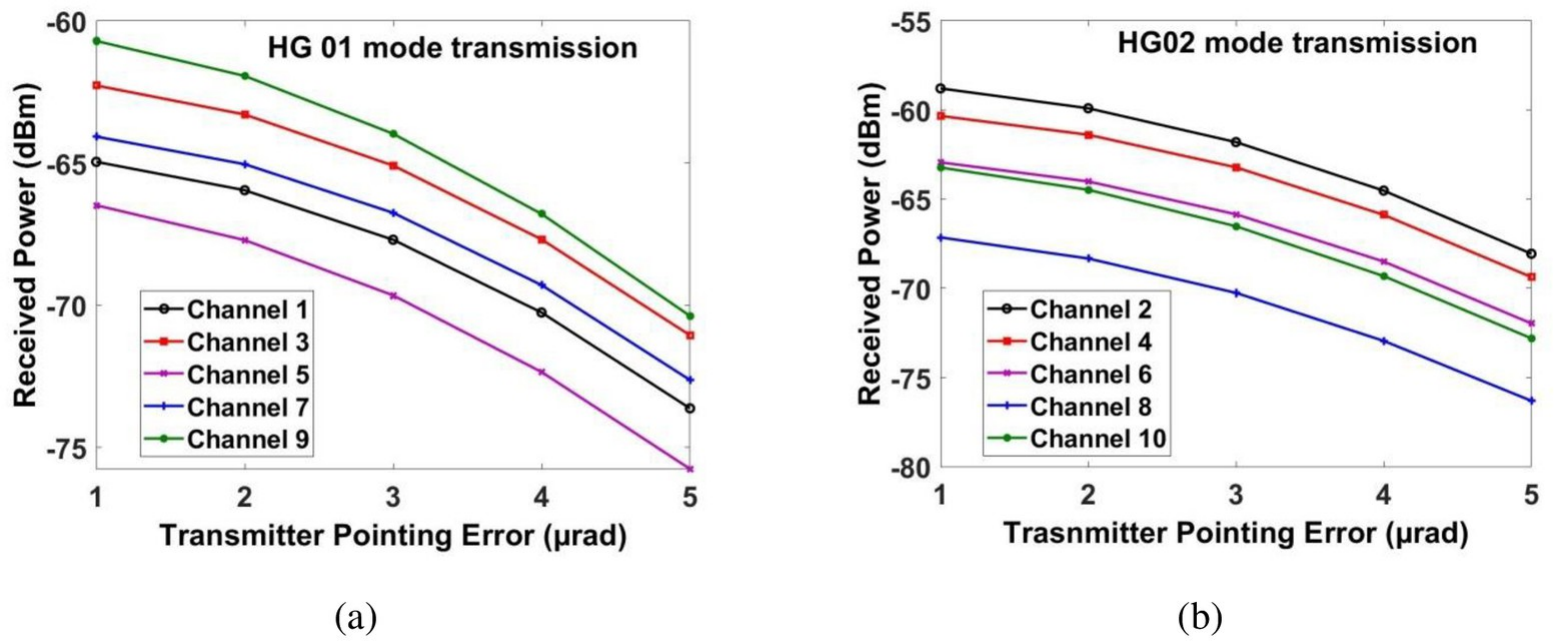
Measured power at output of receiver under the impact of transmitting error for (a) channels 1, 3, 5, 7 and 9 for HG*01* mode transmission and (b) channels 2, 4, 6, 8 and 10 for HG*02* mode transmission.

**Fig 14:**
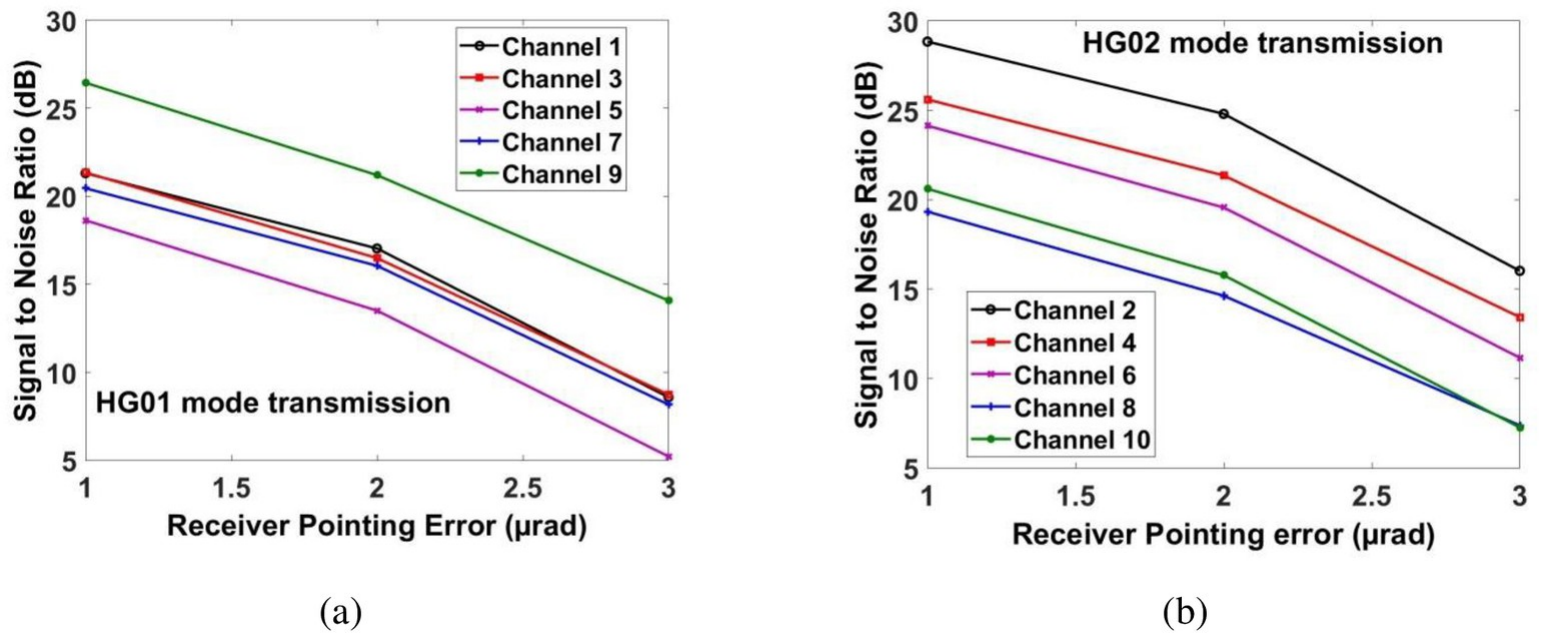
Evaluation of measured SNR under receiving pointing error for (a) channels 1, 3, 5, 7 and 9 for HG*01* mode transmission and (b) channels 2, 4, 6, 8 & 10 for HG*02* mode transmission.

**Fig 15:**
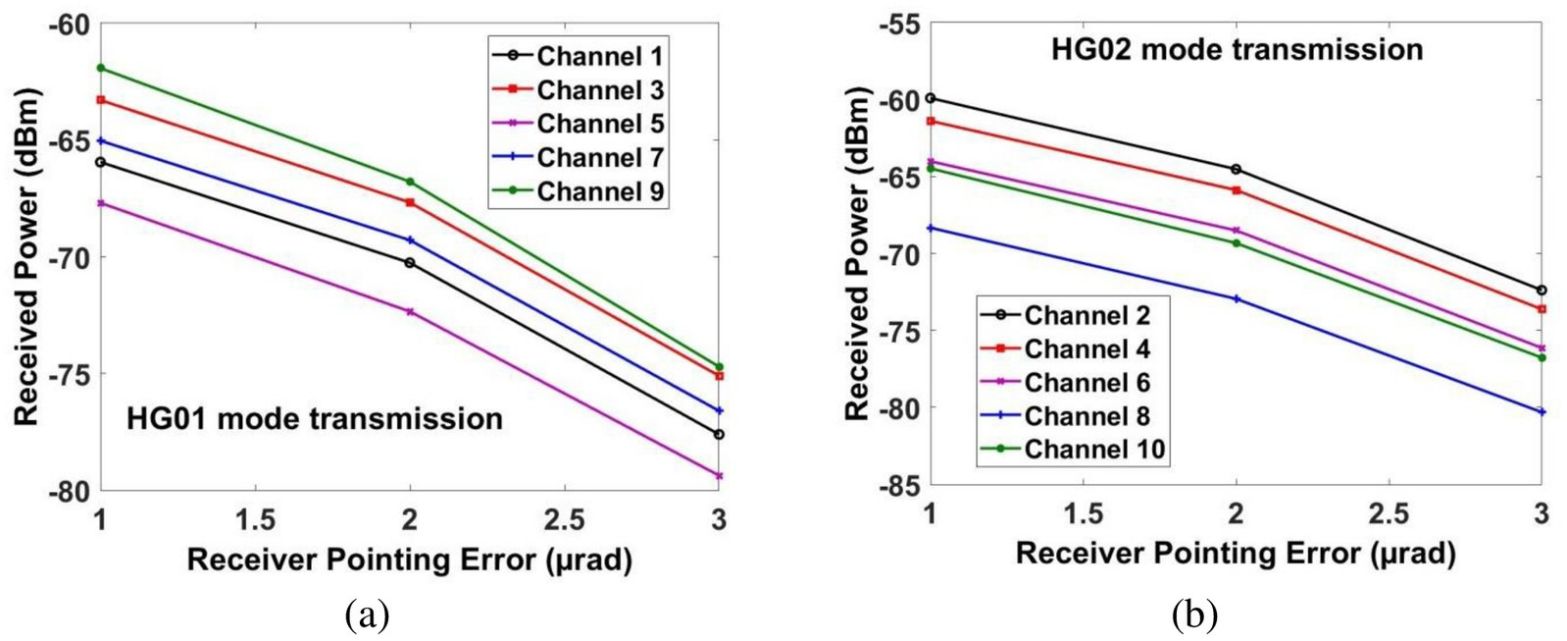
Measured power at output of receiver under the influence of receiving pointing error for (a) channels 1, 3, 5, 7 and 9 for HG*01* mode transmission and (b) channels 2, 4, 6, 8 and 10 for HG*02* mode transmission.

Similarly, Fig 13 shows the measured power for transmitting pointing error. If the transmitting pointing error is less than ≈2.5*μrad*, then all the channels have achieved the required power at the receiver. Beyond that, the power of the recovered signal for all the channels is reduced. Similarly, Figs 14 and 15 show the performance of all the channels under the impact of receiving pointing errors. The figures also show that the impact of receiving pointing errors is more severe as compared to transmitting pointing errors. The acceptable SNR≈20*dB* for all the channels is achieved at the receiving pointing error of 1*μrad*. Similarly, the required power for all the channels is achieved at the receiving pointing error of 1*μrad*. However, channels which are transmitted by using HG*02* mode perform better than the channels which are transmitted by using HG*01* mode.

## IV Conclusion

In this work, we have designed long-reach and terabyte capacity-enabled Is-OWC system by employing the DP-QPSK scheme. The capacity and bandwidth of the proposed Is-OWC system are enhanced by incorporating hybrid MDM and WDM schemes. The hybrid MDM-WDM scheme is incorporated by using modal multiplexing of HG*01* and HG*02* modes to transmit ten channels over 40000kms Is-OWC link. Our results show significant improvement in capacity as well as in bandwidth as compared to previous works reported in the literature [10, 12, 17, 23]. The proposed Is-OWC link can transmit 4*Tbps* data up to 17000*kms* with the required SNR and received power. Moreover, the proposed Is-OWC link can withstand up to 16000*kms* with the transmitting error of 2*μrad* and receiving pointing error of 1*μrad*. This work can further be extended by considering other encoding schemes such as phase shift key (PSK), binary phase shift key (BPSK) etc. as well as real-time experiments for the Is-OWC system.
